# Medical Attention Seeking After Transient Ischemic Attack and Minor Stroke Before and After the UK Face, Arm, Speech, Time (FAST) Public Education Campaign: Results From the Oxford Vascular Study

**DOI:** 10.1001/jamaneurol.2018.1603

**Published:** 2018-07-02

**Authors:** Frank J. Wolters, Linxin Li, Sergei A. Gutnikov, Ziyah Mehta, Peter M. Rothwell

**Affiliations:** 1 Nuffield Department of Clinical Neurosciences, John Radcliffe Hospital, University of Oxford, Headington, Oxford, United Kingdom

## Abstract

**Importance:**

Risk of major stroke is high during the hours and days after transient ischemic attack (TIA) and minor stroke but is substantially reduced by urgent medical treatment. Public education campaigns have improved the response after major stroke, but their association with behavior after TIA and minor stroke is uncertain. The number of potentially preventable early recurrent strokes in patients who delay or fail to seek medical attention is unknown.

**Objective:**

To investigate the association of public education with delays and failure in seeking medical attention after TIA and minor stroke.

**Design, Setting, and Participants:**

Prospective population-based study of all patients with TIA or stroke who sought medical attention between April 1, 2002, and March 31, 2014, registered at 9 general practices in Oxfordshire, United Kingdom. Data analysis took place from July 1, 2013, to March 2, 2015.

**Exposures:**

Face, Arm, Speech, Time (FAST) public education campaign in the United Kingdom.

**Main Outcomes and Measures:**

Number of early recurrent strokes in patients who delayed or failed to seek medical attention, as well as the odds of seeking urgent attention after TIA and minor stroke before vs after initiation of the public education campaign.

**Results:**

Among 2243 consecutive patients with first TIA or stroke (mean [SD] age, 73.6 [13.4] years; 1126 [50.2%] female; 96.3% of white race/ethnicity), 1656 (73.8%) had a minor stroke or TIA. After the FAST campaign, patients with major stroke more often sought medical attention within 3 hours (odds ratio [OR], 2.56; 95% CI, 1.11-5.90; *P* = .03). For TIA and minor stroke, there was no improvement in use of emergency medical services (OR, 0.79; 95% CI, 0.50-1.23; *P* for interaction = .03 vs major stroke) or time to first seeking medical attention within 24 hours (OR, 0.75; 95% CI, 0.48-1.19; *P* for interaction = .006 vs major stroke). Patient perception of symptoms after TIA and minor stroke was associated with more urgent behavior, but correct perception declined after the FAST campaign (from 37.3% [289 of 774] to 27.6% [178 of 645]; OR, 0.64; 95% CI, 0.51-0.80; *P* < .001). One hundred eighty-eight patients had a stroke within 90 days of their initial TIA or stroke, of whom 93 (49.5%) followed unheeded TIAs for which no medical attention was sought, similar before and after the FAST campaign (43 of 538 [8.0%] before vs 50 of 615 [8.1%] after, *P* = .93).

**Conclusions and Relevance:**

This study suggests that in contrast to major stroke, extensive FAST-based public education has not improved the response to TIA and minor stroke in the United Kingdom, emphasizing the need for campaigns that are tailored to transient and less severe symptoms.

## Key Points

**Questions:**

What is the number of potentially preventable early recurrent strokes in the United Kingdom among patients who delay or fail to seek medical attention, and has extensive public education changed patient response?

**Findings:**

Among 2243 consecutive patients with transient ischemic attack or stroke in this population-based study, extensive Face, Arm, Speech, Time (FAST)–based public education has not improved the response to transient ischemic stroke and minor stroke. The percentage of strokes preceded by a transient ischemic attack for which no attention was sought remained unchanged.

**Meaning:**

Public education campaigns tailored to transient and less severe symptoms are needed to encourage urgent patient response to imminent stroke warning signs.

## Teaser

This population-based study investigates the association of public education with improving delays and failure in seeking medical attention among consecutive patients with first transient ischemic attack or stroke.

## Introduction

When left untreated, approximately 5% of patients with transient ischemic attack (TIA) or minor stroke have a major stroke within 24 hours, comprising more than 40% of all recurrent strokes within 30 days.^1^ Urgent investigation and medical treatment substantially reduce risk of early recurrent stroke,^2,3^ as could initial self-medication with aspirin alone.^4^ Consequently, guidelines recommend that patients with high-risk TIA should be assessed urgently.^5,6,7^ However, patients frequently fail to recognize or act on TIA symptoms, either delaying seeking medical attention^8,9^ or not seeking medical attention at all.^10,11^ The number of potentially preventable early recurrent strokes that consequently go unprevented is unknown, although recent public education campaigns designed to increase recognition of major stroke symptoms might change behavior after TIA.

The Face, Arm, Speech, Time (FAST) test was adopted as a tool to improve symptom recognition after stroke^12,13^ and has formed the basis of public education in many countries, including the United Kingdom, Ireland, United States, Australia, and New Zealand, with variants in several non–English-speaking countries. The FAST test was used in an ongoing television public awareness campaign in the United Kingdom from 2009 onward. It appears to have improved the response after major stroke,^14,15,16,17^ but the association of the campaign with patient behavior after TIA and minor stroke has not been determined and may well differ given differences in event duration, severity, and coverage by the FAST acronym.

We prospectively studied patient perception and behavior after TIA and stroke in a population-based study before and during the ongoing FAST campaign. We also investigated the number of early strokes after a TIA for which no medical attention is sought.

## Methods

### Ethical Approval

The Oxford Vascular Study (OxVasc) was approved by the Oxfordshire Research Ethics Committee and included use of routinely collected health care data for investigating incidence rates without consent. Written informed consent or assent was obtained from all participants for additional data.

### Study Design

The OxVasc is a population-based study of all acute vascular events, including TIA and stroke in 92 728 individuals of all ages registered with 100 collaborating primary care physicians at 9 general practices in Oxfordshire, United Kingdom. The OxVasc study methods have been described previously^18^ and are recounted in the online methods (eFigure in the Supplement). The present article includes all consecutive incident TIA and stroke cases, with the exception of subarachnoid hemorrhages, occurring outside the hospital between April 1, 2002, and March 31, 2014. Data analysis took place from July 1, 2013, to March 2, 2015.

Most patients were seen in the dedicated study TIA and stroke clinics or were admitted to the acute stroke service at the principal center (John Radcliffe Hospital, Oxford, United Kingdom) serving the study population. Patients provided informed consent (or assent was obtained from relatives) and were seen by study physicians (among whom were F.J.W., L.L., and P.M.R.) for structured interview using a standard questionnaire as soon as possible after initial presentation to assess their perception about the event and immediate response to symptoms, including date and time of symptom onset, when medical attention was sought and by whom, the first contact with emergency medical services (EMS), and why they did not seek medical attention straightaway in case of delay to medical attention exceeding 3 hours. Patients were routinely questioned about any neurological symptoms within 90 days before their presenting event (ie, unheeded TIAs) and were followed up for recurrent cerebrovascular events (eFigure in the Supplement).^18^ Baseline characteristics, including demographic data, self-reported race/ethnicity, and risk factors for stroke (based on prior diagnosis and current medication use), were recorded, and assessments were made for severity of the event using the National Institutes of Health Stroke Scale. Major stroke was defined as a National Institutes of Health Stroke Scale score exceeding 3. Socioeconomic status was assessed according to the United Kingdom’s 2007 indexes of deprivation.^19^ Based on these indexes, the electoral districts covering our population are less deprived than the rest of the United Kingdom, but still 22% of our districts rank in the lower one-third nationally. Further data were acquired from medical records, ambulance sheets, general practitioner referral letters, and consultation notes. Consent for access to this information was obtained from all participating patients.

To best reflect the response of patients to symptoms, time of symptom onset was defined as time of awaking if onset of stroke was during sleep. In patients unable to call for help, time of symptom onset was considered the moment another person noted their symptoms. Events were classified as FAST positive when at least one symptom from the FAST campaign (ie, facial weakness, arm weakness, or speech disturbance) was present at symptom onset.

### FAST Campaign in the United Kingdom

The initial FAST public education television campaign in the United Kingdom ran from February through April 2009, with 8 weeks of national television broadcasts. With initiation of the 2009 campaign, the *T* in FAST was redesignated “Time to call 999” (ie, EMS) rather than “Test all 3.” Repeated television campaigns ran intermittently for several weeks from October 2009 to early 2010, for 6 weeks in March and April 2011, and for 4 weeks in March 2012, March 2013, and March 2014 and are now continued on a yearly basis. The total campaign investment from 2009 to 2013 was £10.2 million (US $13.6 million). The impact of the campaign as measured by television viewer ratings (1 television viewer rating equals 1% of the target audience of all adults, including multiple views) was 274 in 2012 and 317 in 2013. In October 2005, there was a preceding small-scale 18-month public transport poster campaign.

### Statistical Analysis

Analyses included all first TIA and first stroke occurring outside of the hospital during the study period, with the exception of analyses of patient perception, which excluded patients with reduced consciousness, event-related confusion, or dysphasia. We analyzed patient behavior before April 1, 2009, and after April 1, 2009 (ie, the end of the first major 2-month television campaign) and stratified the analyses (ie, assessed behavior per year of the study) into study years. To assess any association of the FAST campaign with the number of strokes in our study population that could potentially have been prevented by urgent patient behavior after initial symptoms of a TIA, we assessed the number of all 90-day recurrent ischemic strokes preceded by an unheeded TIA. Together with all 90-day recurrences after heeded events, these constitute all early recurrent strokes. We compared the unheeded TIAs with the TIAs in patients who sought medical attention and extrapolated the OxVasc population rates (number of unheeded TIAs divided by the OxVasc source population per 10 years) to the general population to estimate the number of potentially preventable strokes after unheeded TIAs annually.

Time from TIA or stroke symptom onset to first seeking medical attention and the nature of the first medical attention sought—defined as emergency (ie, direct contact with ambulance services or presentation to an emergency department) vs nonemergency (ie, the first contact with a general practitioner or other local health care professional)—were also compared before vs after April 1, 2009, within clinically relevant timing cutoffs of 3 and 24 hours by performing χ^2^ test and computing Mantel-Haenszel test odds ratios (ORs). To further assess time trends, the first contact with EMS vs non-EMS was analyzed per year of the study. Subsequent adjustment was made for time trends and age, sex, race/ethnicity, socioeconomic status, cohabitation, National Institutes of Health Stroke Scale and ABCD2 (age, blood pressure, clinical features of the TIA, duration of symptoms, and history of diabetes) scores, onset during sleep, and occurrence during the weekend^20^ by means of segmented regression analysis,^21^ breaking down time to months and again using April 1, 2009, as the change point. Missing data for covariates in this model were imputed (eTable 1 in the Supplement). We formally assessed for differential associations of the FAST campaign by event type (TIA and minor stroke vs major stroke) by testing for multiplicative interaction in the fully adjusted time-series regression model.

We assessed the association of perception (classified as correct [ie, TIA, stroke, or ministroke] vs incorrect) with subsequent behavior, as well as the proportion of patients with correct initial perception of symptoms before vs after April 1, 2009, in relation to presence of FAST symptoms. Finally, we repeated the trends analyses, comparing only the 5 years immediately before April 1, 2009, vs the subsequent 5 years. All analyses were performed using a software program (SPSS Statistics, version 21.0; IBM). Two-sided α level (type I error) was set at .05.

## Results

Among 2243 consecutive patients with first TIA or stroke in the study period (mean [SD] age, 73.6 [13.4] years; 1126 [50.2%] female; 96.3% of white race/ethnicity), 825 (36.8%) were initially seen with TIA, 831 (37.0%) with minor stroke, and 587 (26.2%) with major stroke. Baseline characteristics of patients (1231 pre-FAST and 1012 post-FAST) are listed in eTable 1 in the Supplement. Data on who sought medical attention were available in 2003 patients (89.3%), and data on first clinician were available in 2196 patients (97.9%). Time from event to call for medical attention and hospital arrival was unknown in 209 patients (9.3%), most commonly because of unconsciousness, dysphasia, dementia, or early death and unavailability of an alternative informant.

### Presence of FAST Symptoms

Of 504 major strokes without collapse or loss of consciousness, 452 (89.7%) had one or more FAST symptoms, as opposed to only 504 of 799 (63.1%) patients initially seen with TIA and 498 of 811 (61.4%) patients with minor stroke. Among patients with minor stroke and TIA, facial weakness was reported in 178 of 780 (22.8%) and 125 of 771 (16.2%), respectively; upper limb motor symptoms in 307 of 785 (39.1%) and 224 of 770 (29.1%), respectively; and speech disturbance in 307 of 810 (37.9%) and 351 of 800 (43.9%), respectively. Among patients with major stroke, 288 of 471 (61.1%) had facial weakness, 377 of 481 (78.4%) had upper limb weakness, and 336 of 458 (73.4%) had speech symptoms.

### Weekend Presentation

**Figure 1.**
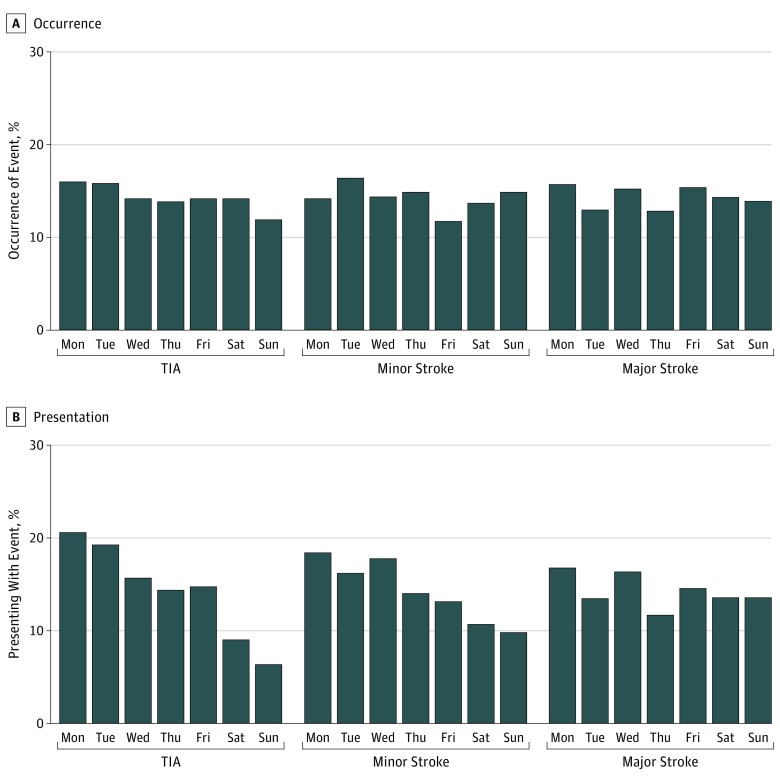
Occurrence of and Presentation With Transient Ischemic Attack (TIA), Minor Stroke, and Major Stroke by Day of the Week Shown is the occurrence of cerebrovascular events, stratified by event severity and day of the week (A), contrasted against the day of the week when medical attention is sought for these events (B).

Patients with TIA or minor stroke were seen less often during the weekend, with higher numbers initially seen directly after the weekend (Figure 1). For major stroke, presentation was similar throughout the week. Weekend presentation of TIA or minor stroke vs major stroke was 267 of 1537 (17.4%) vs 155 of 575 (27.0%) (OR, 0.57; 95% CI, 0.45-0.71; *P* < .001). These findings were similar before and after April 1, 2009.

### Patient Presentation Before and After the FAST Campaign

**Figure 2.**
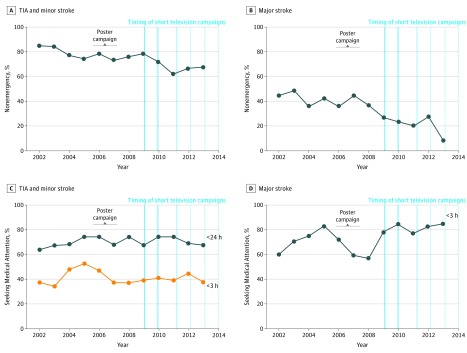
Nonemergency Presentation (Chiefly to a General Practitioner) and Time to Seeking Medical Attention per Year Within the Study Period Shown is nonemergency presentation and time to seeking medical attention for transient ischemic attack (TIA) and minor stroke (A and C) and for major stroke (B and D). The vertical blue lines indicate televised Face, Arm, Speech, Time (FAST) campaigns. The first 2 lines represent 3-month time periods; the third line, 6-week time periods; and the subsequent lines, 4-week time periods.

For major stroke, use of nonemergency services (chiefly a general practitioner) declined steeply from 2009 onward (Figure 2B). Use of EMS was 58.8% before April 1, 2009, vs 78.9% after April 1, 2009 (OR, 2.63; 95% CI, 1.80-3.82; *P* < .001) (Table 1). Moreover, first medical attention was sought more quickly after April 1, 2009, than before April 1, 2009 (Figure 2D). First medical attention was obtained within 3 hours in 67.6% (198 of 293) before April 1, 2009, vs in 81.3% (204 of 251) after April 1, 2009 (OR, 2.08; 95% CI, 1.40-3.11; *P* < .001). Taking into account time trends and potential confounding variables, these changes largely coincided with initiation of the FAST campaign (Table 2). For TIA and minor stroke, medical attention was also sought more often directly via EMS after April 1, 2009, occurring in 21.9% before April 1, 2009, vs in 30.9% after April 1, 2009 (OR, 1.60; 95% CI, 1.28-2.00; *P* < .001) (Table 1). However, this increase was not significantly associated with the FAST campaign in a time-series analysis (Figure 2A). The change was attributable to a baseline trend (OR, 1.01; 95% CI, 1.00-1.02; *P* = .009) rather than to the campaign (adjusted OR, 0.79; 95% CI, 0.50-1.23; *P* = .29) (Table 2). Time to first seeking medical attention after TIA and minor stroke was similar before and after April 1, 2009. Medical attention was obtained within 3 hours in 42.1% (384 of 912) before April 1, 2009, vs in 40.4% (298 of 737) after April 1, 2009 (OR, 0.93; 95% CI, 0.77-1.14; *P* = .49), and was obtained within 24 hours in 70.4% (612 of 869) before April 1, 2009, vs in 70.6% (493 of 698) after April 1, 2009 (OR, 1.01; 95% CI, 0.81-1.26; *P* = .93), and did not change substantially during the course of the study (Table 2 and Figure 2C). The observed association of the FAST campaign with response to TIA and minor stroke differed significantly from the association with response to major stroke for both use of EMS (*P* for interaction in the time-series analysis = .03 vs major stroke) and time to first seeking medical attention within 24 hours (*P* for interaction in the time-series analysis = .006 vs major stroke). Results were similar for TIA and minor stroke and when restricting analyses to 5 years before and 5 years after initiation of the campaign.

**Table 1.** First Health Care Professional Contacted Before and After the 2009 FAST Campaign for TIA and Minor Stroke and for Major Stroke

Variable	No. (%)		OR (95% CI)	*P* Value
	Pre-FAST	Post-FAST		
**TIA and Minor Stroke**	**(n = 897)**	**(n = 735)**		
Nonemergency	701 (78.1)	508 (69.1)	0.63 (0.50-0.78)	<.001
GP	643 (71.7)	447 (60.8)	0.61 (0.50-0.75)	<.001
NHS Direct^a^	21 (2.3)	29 (3.9)	1.71 (0.97-3.03)	.06
Other^b^	37 (4.1)	32 (4.4)	1.06 (0.65-1.72)	.82
Emergency	196 (21.9)	227 (30.9)	1.60 (1.28-2.00)	<.001
A&E	34 (3.8)	51 (6.9)	1.89 (1.21-2.95)	.004
999^c^	143 (15.9)	164 (22.3)	1.51 (1.18-1.94)	.001
Eye hospital	19 (2.1)	12 (1.6)	0.77 (0.37-1.59)	.48
**Major Stroke**	**(n = 308)**	**(n = 256)**		
Nonemergency	127 (41.2)	54 (21.1)	0.38 (0.26-0.56)	<.001
GP	124 (40.3)	47 (18.4)	0.33 (0.23-0.49)	<.001
NHS Direct^a^	1 (0.3)	2 (0.8)	2.42 (0.22-26.81)	.46
Other^b^	2 (0.6)	5 (2.0)	3.05 (0.59-15.84)	.16
Emergency	181 (58.8)	202 (78.9)	2.63 (1.80-3.82)	<.001
A&E	5 (1.6)	9 (3.5)	2.21 (0.73-6.67)	.15
999^c^	176 (57.1)	193 (75.4)	2.30 (1.60-3.30)	<.001

Abbreviations: A&E, accident and emergency department; FAST, Face, Arm, Speech, Time; GP, general practitioner; NHS, National Health Service; OR, odds ratio; TIA, transient ischemic attack.

^a^ Was dissolved in March 2014.

^b^ Includes optician, eye hospital, private physician, medical staff while traveling (eg, airport staff, ship physician, or hotel physician), research physician, or mentioning of symptoms during routine consultant review. Statistical tests comparing pre-FAST vs post-FAST for nonemergency and emergency presentation are interchangeable.

^c^ Emergency medical services.

**Table 2.** Determinants of Urgent Response to TIA and Minor Stroke and to Major Stroke in Segmented Time-Series Analysis

Variable	Use of Emergency Medical Services		Seeking Medical Attention Within 3 h		Seeking Medical Attention Within 24 h	
	OR (95% CI)	*P* Value	OR (95% CI)	*P* Value	OR (95% CI)	*P* Value
**Determinants of Urgent Response to TIA and Minor Stroke**						
Constant	0.06 (0.02-0.14)	<.001	0.25 (0.10-0.64)	.004	0.59 (0.23-1.53)	.28
Baseline trend	1.01 (1.00-1.02)	.009	1.00 (0.99-1.01)	.87	1.01 (1.00-1.01)	.03
Change at intervention^a^	0.79 (0.50-1.23)	.29	0.87 (0.58-1.29)	.48	0.75 (0.48-1.19)	.22
Trend after intervention	1.01 (0.99-1.02)	.42	1.00 (0.99-1.01)	.59	1.00 (0.99-1.01)	.51
Age	1.00 (0.99-1.01)	.64	1.01 (1.00-1.02)	.03	1.01 (1.00-1.02)	.22
Female sex	0.89 (0.70-1.12)	.31	0.89 (0.72-1.09)	.26	0.84 (0.67-1.06)	.13
Nonwhite race/ethnicity	1.28 (0.64-2.56)	.47	0.76 (0.38-1.52)	.43	0.85 (0.46-1.57)	.60
IMD	1.01 (0.99-1.02)	.50	1.01 (0.99-1.02)	.42	1.01 (0.99-1.03)	.40
Living alone	1.17 (0.89-1.52)	.26	0.81 (0.64-1.03)	.08	0.92 (0.70-1.19)	.51
ABCD2 score	1.22 (1.13-1.33)	<.001	1.22 (1.13-1.30)	<.001	1.31 (1.21-1.42)	<.001
Symptoms on awaking	0.78 (0.59-1.04)	.09	0.72 (0.56-0.93)	.01	0.83 (0.63-1.11)	.20
Weekend occurrence	0.99 (0.76-1.27)	.90	0.80 (0.64-1.00)	.05	0.53 (0.42-0.68)	<.001
**Determinants of Urgent Response to Major Stroke**						
Constant	0.60 (0.09-3.68)	.43	0.28 (0.03-2.52)	.26	NA	NA
Baseline trend	1.01 (1.00-1.02)	.15	0.99 (0.98-1.00)	.17	NA	NA
Change at intervention^a^	1.68 (0.76-3.75)	.15	2.56 (1.11-5.90)	.03	NA	NA
Trend after intervention	1.00 (0.98-1.02)	.82	1.01 (0.99-1.04)	.31	NA	NA
Age	0.98 (0.97-1.00)	.07	1.01 (0.99-1.03)	.30	NA	NA
Female sex	0.86 (0.57-1.29)	.47	1.04 (0.68-1.60)	.85	NA	NA
Nonwhite race/ethnicity	1.27 (0.42-3.82)	.67	3.28 (0.66-16.39)	.15	NA	NA
IMD	1.00 (0.97-1.03)	.82	0.98 (0.95-1.02)	.31	NA	NA
Living alone	1.58 (1.01-2.46)	.05	0.60 (0.36-1.00)	.05	NA	NA
Stroke severity on NIHSS	1.16 (1.11-1.22)	<.001	1.10 (1.06-1.15)	<.001	NA	NA
Symptoms on awaking	0.79 (0.50-1.24)	.31	0.52 (0.30-0.88)	.02	NA	NA

Abbreviations: ABCD, age, blood pressure, clinical features of the TIA, duration of symptoms, and history of diabetes; FAST, Face, Arm, Speech, Time; IMD, Index of Multiple Deprivation (indicating socioeconomic status, with higher indexes indicating more deprived); NA, not applicable; NIHSS, National Institutes of Health Stroke Scale; OR, odds ratio; TIA, transient ischemic attack.

^a^ The odds ratios for change at intervention reflect the change at the infliction point (ie, the start of the FAST campaign) in use of emergency medical services or time to seeking medical attention, adjusted for all other variables, including trends before and after campaign initiation. For example, in the penultimate right column, the odds of seeking medical attention within 24 hours were 0.75 times lower after initiation of the campaign than before.

### Stroke After Unheeded TIAs

Ninety-five patients who had sought attention for an initial TIA or stroke had a first or recurrent stroke by 90-day follow-up. In addition, 93 patients with stroke reported having symptoms of a TIA for which they had not sought medical attention during the 90 days before their presenting stroke. Therefore, there were 188 early strokes after initial TIA or stroke in all patients, in whom 93 (49.5%) occurred after a TIA for which no medical attention was sought. This number of strokes preceded by an unheeded TIA was similar before and after the FAST campaign (43 of 538 [8.0%] before vs 50 of 615 [8.1%] after, *P* = .93). The median interval from the first unheeded TIA to a stroke was 6 days (interquartile range [IQR], 1-17 days). The median duration of unheeded TIAs was 20 minutes (IQR, 5-60 minutes). Thirty-two of 93 patients (34.4%) with unheeded TIAs preceding their stroke had more than one TIA before stroke occurrence. Among all 93 patients with unheeded TIAs preceding stroke, 69 (74.2%) were not taking antiplatelet medication or oral anticoagulants at the time of their stroke. Compared with TIAs for which medical attention was sought, unheeded TIAs were shorter, more often consisted of a single symptom only, and were less likely to disturb speech (Table 3). Only 34.8% of those with unheeded TIAs had any symptom covered by the FAST acronym. Patients with unheeded TIAs were also younger, were more often male, resided in more socially deprived neighborhoods, and lived in a shared household. Most recurrent stroke after heeded TIA occurred despite specialized hospital care within 24 hours (67.4% [58 of 86 for whom data were available]) or 72 hours (87.2% [75 of 86]) of the initial event.

**Table 3.** Patient and Event Characteristics of Those Initially Seen With TIA vs Those Initially Seen With Stroke After an Unheeded TIA^a^

Variable	All TIAs (N = 825)	Unheeded TIAs (n = 93)	*P* Value
Age, mean (SD), y	72.7 (13.4)	67.7 (14.9)	<.001
Female sex, No. (%)	426 (51.6)	38 (40.9)	.05
Nonwhite race/ethnicity, No. (%)	22 (3.2)	2 (2.2)	.63
IMD, median, (IQR)	7.2 (4.8-13.1)	9.4 (5.0-13.0)	.08
Living alone, No. (%)	221 (28.2)	16 (17.8)	.04
Increased cardiovascular risk, No. (%)^b^	26 (28.6)	236 (28.8)	.97
Duration of TIA, median (IQR), min	30 (10-180)	20 (5-60)	.03
Isolated symptoms, No. (%)^c^	401 (50.2)	69 (75.8)	<.001
Symptom type, No. (%)^d^			
Motor	340 (42.7)	33 (35.9)	.35
Facial weakness	125 (16.2)	3 (3.4)	.05
Arm weakness	224 (29.1)	18 (19.4)	.72
Sensory	209 (26.3)	19 (20.7)	.37
Speech	349 (43.7)	16 (17.2)	<.001
Visual	231 (29.1)	28 (30.1)	.92
Vertigo	48 (6.0)	8 (8.6)	.15
FAST positive, No. (%)	504 (63.1)	32 (34.8)	<.001

Abbreviations: FAST, Face, Arm, Speech, Time; IMD, Index of Multiple Deprivation (indicating socioeconomic status, with higher indexes indicating more deprived); IQR, interquartile range; TIA, transient ischemic attack.

^a^ For the percentages, some denominators vary from the heading totals because of missing data.

^b^ Presence of at least 2 vascular risk factors (ie, hypertension, diabetes, hypercholesterolemia, or current smoking).

^c^ One type of symptoms present (eg, sensory symptoms only).

^d^ *P* values adjusted for co-occurrence of any other symptoms.

### Patient Perception of Symptoms

In 1419 patients without severe speech disturbance, cognitive impairment, or event-related confusion after TIA or minor stroke who provided their initial perception of symptoms, 467 (32.9%) correctly attributed these to TIA or stroke. Patients with correct perception sought medical attention more quickly (241 of 438 [55.0%] when correct vs 337 of 887 [38.0%] when incorrect within 3 hours; OR, 1.45; 95% CI, 1.18-1.77; *P* < .001) and were more likely to directly contact EMS (130 of 458 [28.4%] when correct vs 193 of 946 [20.4%] when incorrect; OR, 1.55; 95% CI, 1.20-2.00; *P* = .001) (eTable 2 in the Supplement). Correct perception of symptoms after TIA and minor stroke was less common after April 1, 2009, compared with before April 1, 2009 (from 37.3% [289 of 774] to 27.6% [178 of 645]; OR, 0.64; 95% CI, 0.51-0.80; *P* < .001), particularly in FAST-positive cases (eTable 3 in the Supplement). Decline in correct perception was seen for both TIA and minor stroke and was similar for low-risk and high-risk TIA. Among 245 of 588 patients (41.7%) with major stroke who provided their initial perception of symptoms, perception was correct in 116 patients (47.3%) but was not associated with the proportion contacting EMS (54.5% [61 of 112] when correct vs 53.6% [67 of 125] when incorrect; OR, 1.04; 95% CI, 0.62-1.73; *P* = .89) or time to seeking first medical attention (median, 60 minutes [IQR, 10-173 minutes] when correct vs 60 minutes [IQR, 15-537 minutes] when incorrect; *P* = .12). In most major strokes (483 of 543 [89.0%]), first medical attention was sought by someone other than the patient. For TIA and minor stroke, 52.5% (767 of 1460) of patients did not call for medical aid themselves but relied on their spouse (373 [25.5%]), another relative or close acquaintance (262 [17.9%]), a bystander (63 [4.3%]), or staff at a care home (61 [4.2%]). Seventy-five of 1460 patients (5.1%) did not report symptoms to a health care professional until a routine appointment with a physician. The proportion of patients seeking medical attention themselves was similar for TIA and minor stroke and was unaltered before vs after April 1, 2009. Data on the initial diagnostic impression of relatives or bystanders were not collected.

### Reasons for Delay in Seeking Medical Attention

Of 864 patients with TIA or minor stroke who delayed seeking medical attention more than 3 hours, 654 (75.7%) provided a reason, usually attributing symptoms to another (less harmful) cause (157 [24.0%]), being reassured by or awaiting improvement of symptoms (153 [23.4%]), not being worried about initial symptoms (145 [22.2%]), or not seeking medical attention because symptom onset occurred outside of office hours (50 [7.6%]) (eTable 4 in the Supplement). Reasons for delay were similar for TIA and minor stroke (eTable 4 in the Supplement) and were essentially unchanged before and after April 1, 2009.

## Discussion

In contrast to major stroke, we found in this study that for TIA and minor stroke the UK nationwide televised FAST campaign has not improved patient use of EMS or patient delay in seeking medical attention. Moreover, the percentage of strokes that followed shortly after an initial TIA for which no medical attention was sought remained unchanged after the FAST campaign, representing approximately 100 potentially preventable strokes per 1 million inhabitants annually.

It is likely that many individuals experience transient neurological symptoms for which they do not seek medical attention and do not have stroke. The results of large randomly dialed telephone studies^10,11^ have suggested that approximately 15% of people in the general population had experienced transient neurological symptoms during the previous few years, such as weakness, numbness, or visual problems. Although we cannot determine the total number of unheeded TIAs in our study population, we found that the share of strokes preceded by symptoms suggestive of TIA in the community for which no medical attention is sought has remained unchanged after the FAST campaign, affirming that these ignored events are an important target for stroke prevention. The large number of patients with TIA during the weekend who seek medical attention only days later during office hours emphasizes that less severe or shorter-lasting symptoms are not thought to require immediate medical attention, with no reduction since the FAST campaign. Seventy-five percent (70 of 93) of patients with stroke after unheeded TIAs were not taking antithrombotic medication, highlighting the missed opportunity for secondary prevention.^2,3,4^ Given the high effectiveness of preventive strategies within the first hours and days after TIA,^4^ the influence of public education will depend largely on its ability to convince patients to take action within this crucial window of time.

Use of EMS has repeatedly been shown to be one of the most important factors in early hospital arrival after TIA and minor stroke.^8,9,22,23^ We observed no positive association of the FAST television campaign with presentation via EMS after TIA and minor stroke. Even when patients attributed their symptoms to stroke, still less than one-third (130 of 458) contacted EMS, which is in line with the findings of a 2012 UK survey.^24^ Studies^14,15,16,17^ have indicated a moderate influence of public education on presentation after stroke, but no published studies to date have assessed the effect of large public education campaigns on presentation after TIA and minor stroke. Compared with major stroke, one of the main differences we found in the response to TIA and minor stroke is the high association of symptom recognition with subsequent behavior in the latter. While severe symptoms require medical aid regardless of their cause, transient or minor symptoms may leave room for deliberation and attribution of symptoms to other (less worrisome) causes. A population-based survey in the United Kingdom recently showed that, despite recall of the FAST campaign, recognition and the response to hypothetical stroke scenarios did not improve.^25^ The decline in correct recognition of symptoms that we observed after extensive FAST-based public education campaigns suggests that patients may be falsely reassured when their symptoms do not match the more severe symptoms depicted in public education advertisements.^26^ The limited sensitivity of the FAST acronym for TIA and minor stroke (approximately 60% vs 95% for major stroke based on the Results section herein) may further explain the lack of positive influence of the campaign in this group of patients and emphasizes the need for adapted forms of public education.

### Limitations

Although we believe that our findings are valid and should guide future public education, there are some limitations. First, the retrospective diagnosis of unheeded TIAs preceding stroke is inevitably subjective and may be subject to recall bias. However, we applied the same diagnostic criteria that we apply when making a diagnosis acutely after a TIA, and we will have underestimated unheeded TIAs preceding major stroke in patients who were unable to report these because of drowsiness, dysphasia, or dementia. Moreover, although we allowed a 90-day cutoff for preceding TIA to limit recall bias of distant events, most TIAs occurred within the week before the stroke and mirrored previous prospective investigations of the natural history of stroke risk after TIA.^1^ Second, we did not record the diagnostic perception of bystanders or relatives, nor did we directly ask about individual exposure to and awareness of the campaign. Third, increased but slow presentation of otherwise nonpresenters after the FAST campaign may have biased time trends of TIA and minor stroke toward the null. Fourth, in view of the small fraction of people in our study who were of nonwhite race/ethnicity, our results may not be fully applicable to racial/ethnic minorities, who have previously been reported to delay to presentation longer.^8^

## Conclusions

In our study, a lack of improvement in patient response to TIA and minor stroke was found after extensive FAST-based public education television campaigns in the United Kingdom. This highlights the need for effective public education to be tailored to transient and minor stroke symptoms, as well as major stroke.

## Notes

Supplement. **eFigure.** Flow Chart of Case Ascertainment in OXVASC **eTable 1.** Baseline Characteristics of Patients With a First in Study TIA or Stroke **eTable 2.** Delay in Presentation and Choice of First Contact With Healthcare Services in Relation to Patients’ Perception of Symptoms in TIA and Minor Stroke **eTable 3.** Patient Perception of TIA and Minor Stroke Symptoms Before and After Initiation of the FAST Campaign **eTable 4.** Reasons for More Than 3 Hour Delay in Seeking Medical Attention
