# Supplementary material for: Medical Attention Seeking After Transient Ischemic Attack and Minor Stroke Before and After the UK Face, Arm, Speech, Time (FAST) Public Education Campaign: Results From the Oxford Vascular Study
Source: JAMA Neurol. 2018 Jul 2;75(10):1225–33. doi: 10.1001/jamaneurol.2018.1603 (PMC6233848; doi:10.1001/jamaneurol.2018.1603)
Supplement: Supplement. — eFigure. Flow Chart of Case Ascertainment in OXVASC eTable 1. Baseline Characteristics of Patients With a First in Study TIA or Stroke eTable 2. Delay in Presentation and Choice of First Contact With Healthcare Services in Relation to Patients’ Perception of Symptoms in TIA and Minor Stroke eTable 3. Patient Perception of TIA and Minor Stroke Symptoms Before and After Initiation of the FAST Campaign eTable 4. Reasons for More Than 3 Hour Delay in Seeking Medical Attention [file jamaneurol-75-1225-s001.pdf]

Supplementary Online Content

Wolters FJ, Li L, Gutnikov SA, Mehta Z, Rothwell PM. Medical attention seeking after transient ischemic attack and minor stroke in relation to the UK Face, Arm, Speech, Time (FAST) public education campaign: results from the Oxford Vascular Study. *JAMA Neurol*. Published online July 2, 2018. doi:10.1001/jamaneurol.2018.1603

eFigure. Flow Chart of Case Ascertainment in OXVASC

eTable 1. Baseline Characteristics of Patients With a First in Study TIA or Stroke

eTable 2. Delay in Presentation and Choice of First Contact With Healthcare Services in Relation to Patients' Perception of Symptoms in TIA and Minor Stroke

eTable 3. Patient Perception of TIA and Minor Stroke Symptoms Before and After Initiation of the FAST Campaign

eTable 4. Reasons for More Than 3 Hour Delay in Seeking Medical Attention

This supplementary material has been provided by the authors to give readers additional information about their work.

© 2018 Wolters et al. *JAMA Neurology.*

eFigure. Flow Chart of Case Ascertainment in OXVASC

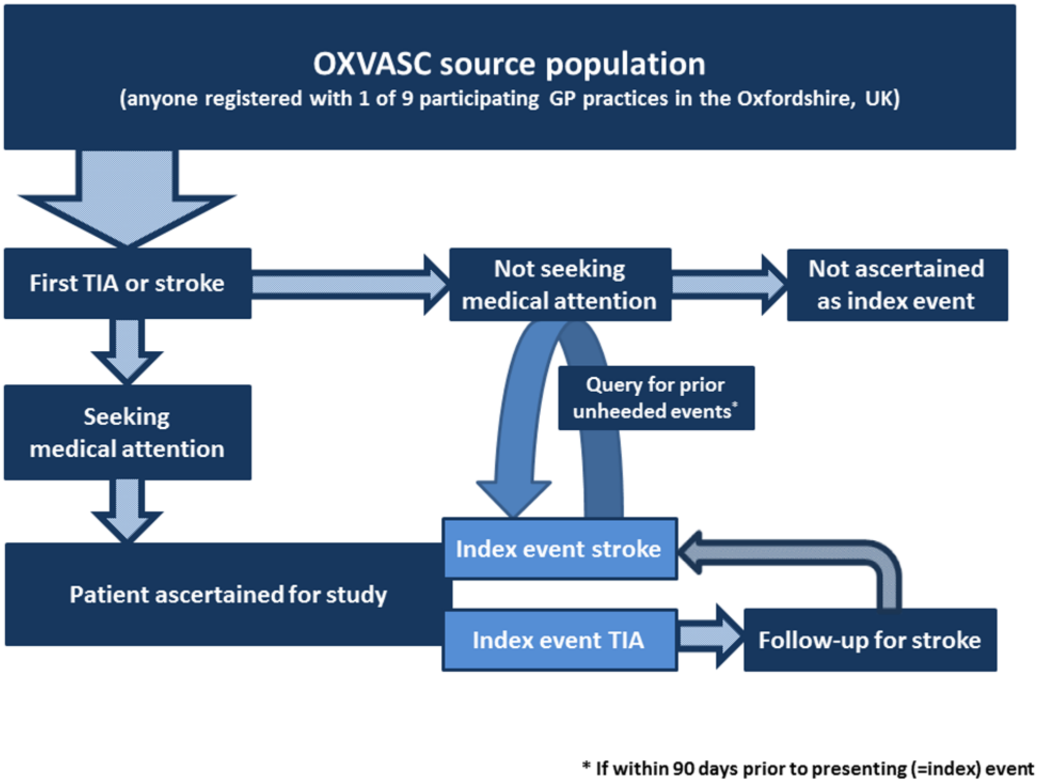

The Oxford Vascular Study (OXVASC) is a population-based study of all acute vascular events, including stroke and TIA in 92,728 individuals of all ages registered with 100 collaborating primary care physicians in nine general practices in the Oxfordshire, United Kingdom. Multiple overlapping methods are used to achieve near complete ascertainment of all care-seeking individuals with stroke or TIA (defined in accordance with WHO and NINDS criteria as rapidly developed clinical sign of focal or global disturbance of cerebral function, lasting less than 24 hours, with no apparent non-vascular cause). These include: 1) a daily TIA and stroke clinic to which participating general practitioners (GPs) and the local emergency department refer individuals with suspected TIA or stroke whom they would not normally admit directly to hospital; 2) daily searches of admissions to hospital wards and emergency department attendance; 3) monthly searches of GP diagnostic coding and hospital discharge codes; 4) monthly searches of all carotid imaging studies performed in local hospitals; 5) daily contact with hospital bereavement officers to identify patients brought into hospital dead or who died soon after arrival, and review of all death certificates in the study practices and ICD10 vascular death codes from the local Department of Public Health. A flow chart of case ascertainment procedure is presented above. The John Radcliffe Hospital serves as the primary referral site for acute stroke care in the study area, and the study TIA clinic receives >95% of outpatient referrals with TIA in the study population.

© 2018 Wolters et al. *JAMA Neurology.*

eTable 1. Baseline Characteristics of Patients With a First in Study TIA or Stroke

	**Pre-FAST** (n=1231)	**Post-FAST** (n=1012)	**P-value**
Age (mean±SD)	74.1 (±12.9)	73.1 (±13.9)	0.07
Female sex	638 (51.8)	488 (48.2)	0.09
Caucasian ethnicity	1093 (96.5)	747 (96.0)	0.61
Past medical history			
Hypertension	668 (55.9)	564 (56.3)	0.85
Diabetes	138 (11.5)	148 (14.8)	0.02
Hyperlipidaemia	385 (34.5)	295 (33.9)	0.78
Current smoking	188 (15.6)	152 (15.5)	0.98
Atrial fibrillation	218 (18.3)	187 (18.7)	0.81
Myocardial infarction	88 (7.6)	94 (9.4)	0.13
Peripheral vascular disease	84 (7.0)	42 (4.2)	0.005
Socioeconomic status, IMD* (median, IQR)	7.6 (4.8-13.0)	7.6 (4.7-13.1)	0.76
Presenting study TIA/stroke			
NIHSS (mean±SD)**	5.0 (±6.7)	5.3 (±7.1)	0.59
$ABCD^2$ ≥4***	246 (58.6)	179 (54.1)	0.21
Duration in minutes (median, IQR)***	30 (10-150)	30 (10-180)	0.91
Weekend occurrence	347 (28.2)	267 (26.4)	0.34

SD = standard deviation; * Index of multiple deprivation – a higher score reflects a higher level of deprivation; NIHSS = National Institutes of Health stroke scale; IQR = interquartile range; ** stroke only; *** TIA only

Characteristics are presented of the non-imputed data. Missing data were imputed using fivefold multiple imputation. This (Bayesian) procedure creates multiple copies of the dataset, replacing the missing values by imputed values on the basis of the observed data. Standard statistical methods are then applied to fit the model of interest to each of the imputed datasets, and subsequently averaged to give overall estimated associations. Of covariates included in the regression models, we had missing data for ethnicity (14.8%), socioeconomic status (8.7%), cohabiting (5.4%), NIHSS/$ABCD^2$ scores (4.9%), and onset during sleep (12.2%).

© 2018 Wolters et al. *JAMA Neurology.*

eTable 2. Delay in Presentation and Choice of First Contact With Healthcare Services in Relation to Patients' Perception of Symptoms in TIA and Minor Stroke

	Correct perception (%)	Incorrect perception (%)	OR [95% CI]	p-value
**Time to seeking medical aid**				
<3 hours	241 (55.0)	337 (38.1)	REFERENCE	–
3-24 hours	101 (23.1)	255 (28.8)	0.55 [0.42-0.74]	<0.001
1-3 days	57 (13.0)	140 (15.8)	0.57 [0.40-0.81]	0.001
≥3 days	39 (8.9)	153 (17.3)	0.36 [0.24-0.53]	<0.0001
**First medical aid**				
Emergency	130 (28.4)	193 (20.4)	1.55 [1.20-2.00]	0.001
Non-emergency	328 (71.6)	689 (79.6)		

© 2018 Wolters et al. *JAMA Neurology.*

eTable 3. Patient Perception of TIA and Minor Stroke Symptoms Before and After Initiation of the FAST Campaign

	Pre-FAST (%)		Post-FAST (%)		Post- versus pre-FAST	
	Correct (%)	Incorrect (%)	Correct (%)	Incorrect (%)	**OR [95% CI]**	**P-value**
**TIA & minor stroke**	289 (37.3)	485 (62.7)	178 (27.6)	467 (72.4)	0.64 [0.51-0.80]	0.0001
FAST-negative	60 (23.0)	201 (77.0)	49 (18.5)	216 (81.5)	0.76 [0.50-1.16]	0.20
FAST-positive	220 (44.9)	270 (55.1)	129 (34.5)	245 (65.5)	0.65 [0.49-0.85]	0.002
**TIA**	149 (39.3)	230 (60.7)	100 (30.7)	226 (69.3)	0.68 [0.50-0.93]	0.02
**Minor stroke**	140 (35.4)	255 (64.6)	78 (24.5)	241 (75.5)	0.59 [0.43-0.82]	0.002
**TIA**						
$ABCD^2<4$	51 (33.8)	100 (66.2)	37 (27.4)	98 (72.6)	0.74 [0.45-1.23]	0.24
$ABCD^2\geq4$	88 (41.7)	123 (58.3)	45 (30.4)	103 (69.6)	0.61 [0.39-0.95]	0.03
**Isolated symptoms**						
Motor	54 (57.4)	40 (42.6)	20 (32.8)	41 (67.2)	0.36 [0.18-0.71]	0.003
Speech	24 (32.9)	49 (67.1)	19 (31.1)	42 (68.9)	0.92 [0.45-1.92]	0.83
Other	34 (20.1)	135 (79.9)	31 (17.0)	151 (83.0)	0.81 [0.48-1.40]	0.46

OR= odds ratio; CI = confidence interval.

Patient perception of the event was classified correct, when the symptoms were attributed to ‘stroke’, ‘mini-stroke’, or ‘TIA’. Isolated symptoms refer to events with only one type of focal symptom present (i.e. motor, sensory, speech, visual, or vertigo)

© 2018 Wolters et al. *JAMA Neurology.*

eTable 4. Reasons for More Than 3 Hour Delay in Seeking Medical Attention

TIA & Minor stroke (n=654)		TIA (n=306)		Minor ischaemic stroke (n=311)	
**Reason for delay**	**N (%)**	**Reason for delay**	**N (%)**	**Reason for delay**	**N (%)**
Other self-diagnosis	157 (24.0)	(Awaiting) improvement	83 (27.1)	Other self-diagnosis	101 (29.0)
(Awaiting) improvement	153 (23.4)	Not worried	65 (21.2)	Not worried	80 (23.0)
Not worried	145 (22.2)	Other self-diagnosis	56 (18.3)	(Awaiting) improvement	70 (20.1)
Out-of-hours	50 (7.6)	Out-of-hours	26 (8.5)	Out-of-hours	24 (6.9)
Pending appointment	30 (4.6)	Pending appointment	23 (7.5)	No idea about the cause	18 (5.2)
No idea about the cause	29 (4.4)	No idea about the cause	11 (3.6)	Not to bother others	10 (2.9)
Not to bother others	20 (3.1)	Not to bother others	10 (3.3)	Pending appointment	7 (2.0)
Other	70 (10.7)	Other	32 (10.5)	Other	38 (10.9)

© 2018 Wolters et al. *JAMA Neurology.*
